# Thermal Ablation Combined with Immune Checkpoint Blockers: A 10-Year Monocentric Experience

**DOI:** 10.3390/cancers16050855

**Published:** 2024-02-21

**Authors:** Baptiste Bonnet, Louis Tournier, Frédéric Deschamps, Steven Yevich, Aurélien Marabelle, Caroline Robert, Laurence Albiges, Benjamin Besse, Victoire Bonnet, Thierry De Baère, Lambros Tselikas

**Affiliations:** 1 Gustave Roussy, Département d’Anesthésie, Chirurgie et Interventionnel (DACI), F-94805 Villejuif, France; frederic.deschamps@gustaveroussy.fr (F.D.); thierry.debaere@gustaveroussy.fr (T.D.B.); lambros.tselikas@gustaveroussy.fr (L.T.); 2 Department of Radiology, Saint-Louis Hospital, Université de Paris, F-75010 Paris, France; louis.tournier@aphp.fr; 3 Department of Interventional Radiology, Division of Diagnostic Imaging, The University of Texas MD Anderson Cancer Center, Houston, TX 77030, USA; syevich@mdanderson.org; 4 Drug Development Department (DITEP), F-94805 Villejuif, France; aurelien.marabelle@gustaveroussy.fr; 5 Laboratoire de Recherche Translationnelle en Immunothérapies (LRTI), Inserm U1015, F-94805 Villejuif, France; 6 Faculty of Medicine, Paris-Saclay University, F-94276 Le Kremlin Bicêtre, France; 7 Gustave Roussy, Département de Médecine Oncologique, F-94805 Villejuif, France; caroline.robert@gustaveroussy.fr (C.R.); laurence.albiges@gustaveroussy.fr (L.A.); benjamin.besse@gustaveroussy.fr (B.B.); 8 Medicine Department, Campus Pierre et Marie Curie, Sorbonne University, 4 Place Jussieu, F-75005 Paris, France; victoire.bonnet@etu.sorbonne-universite.fr

**Keywords:** ablation, percutaneous, immunotherapy, immune checkpoint inhibitors, drug-related side effects and adverse reactions, safety

## Abstract

**Simple Summary:**

A 10-year experience in cancer therapy using concomitant treatment of immunotherapy and percutaneous thermal ablation is shared in this article. Based on a retrospective cohort of 78 patients, the feasibility, safety and efficacy of such combined treatments were assessed. Most patients received immune checkpoint blocker monotherapy combined with radiofrequency or cryotherapy ablation. The feasibility and safety profile were found to be excellent, with complications equivalent to those reported when each treatment was performed separately. Overall, thermal ablation outcomes were found to be similar to standards for patients not on immunotherapy.

**Abstract:**

Purpose: We report a 10-year experience in cancer therapy with concomitant treatment of percutaneous thermal ablation (PTA) and immune checkpoint blockers (ICBs). Material and methods: This retrospective cohort study included all patients at a single tertiary cancer center who had received ICBs at most 90 days before, or 30 days after, PTA. Feasibility and safety were assessed as the primary outcomes. The procedure-related complications and immune-related adverse events (irAEs) were categorized according to the Common Terminology Criteria for Adverse Events v5.0 (CTCAE). Efficacy was evaluated based on overall survival (OS), progression-free survival (PFS), and local progression-free survival (_L_PFS) according to the indication, ablation modality, neoplasm histology, and ICB type. Results: Between 2010 and 2021, 78 patients (57% male; median age: 61 years) were included. The PTA modality was predominantly cryoablation (CA) (61%), followed by radiofrequency ablation (RFA) (31%). PTA indications were the treatment of oligo-persistence (29%), oligo-progression (14%), and palliation of symptomatic lesions or prevention of skeletal-related events (SREs) (56%). Most patients received anti-PD1 ICB monotherapy with pembrolizumab (*n* = 35) or nivolumab (*n* = 24). The feasibility was excellent, with all combined treatment performed and completed as planned. Ten patients (13%) experienced procedure-related complications (90% grade 1–2), and 34 patients (44%) experienced an irAE (86% grade 1–2). The only factor statistically associated with better OS and PFS was the ablation indication, favoring oligo-persistence (*p* = 0.02). Tumor response was suggestive of an abscopal effect in four patients (5%). Conclusions: The concomitant treatment of PTA and ICBs within 2–4 weeks is feasible and safe for both palliative and local control indications. Overall, PTA outcomes were found to be similar to standards for patients not on ICB therapy. While a consistently reproducible abscopal effect remains elusive, the safety profile of concomitant therapy provides the framework for continued assessment as ICB therapies evolve.

## 1. Introduction

Over the past decade, the development of anti-cancer immunotherapies in routine care has led to a spectacular improvement in the survival of patients with advanced cancers [1,2]. Immunotherapies indirectly interfere with tumor cell growth by boosting the anti-tumor immune response. Recently, the most studied ICBs have been ones that act upon anti-cytotoxic T-lymphocyte-associated protein 4 (anti-CTLA-4) and anti-programmed cell death (ligand) protein 1 (anti-PDL1 and anti PDL-1) [3,4]. Widely approved for many tumor types, their use has shown an indisputable increase in patient survival in various tumoral subtypes [5,6]. 

However, a pronounced long-term benefit still only manifests in a minority of patients [7,8]. Moreover, the increasing use and combination of ICB treatments has increased the risks of immune-related adverse events (irAEs). IrAEs present with a wide spectrum, from mild inflammatory to disabling or life-threatening sequela, and they can limit the use of ICBs with dose modulation or intermittent holidays [9]. The realities of irAEs and the uncertainty of long-term benefits of ICBs stress the need to continue with the evaluation of combined treatments between systemic ICBs and local treatment alternatives.

Local treatment alternatives have been developed to target specific sites of tumors, and several minimally invasive options have been rapidly advanced over the past decade. Indications range from curative treatment of small, isolated primary tumors or oligo-metastatic disease to palliative relief of tumor-associated pain and prevention of complications [10,11]. Two minimally invasive modalities that have developed in tandem are radiation therapy and percutaneous thermal ablation (PTA). Both function through different applications of energy, the former by focusing radiation energy to damage tumor cells and the latter by applying radiological image guidance to guide a needle into the tumor before administering thermal energy (heat or cold) at the needle tip to damage tumor cells. 

In the context of combination with immunotherapies, both radiation therapy and PTA have raised hopes of achieving a synergistic effect [12]. Radiation therapy has recently been shown to stimulate the antitumor immune response, which can synergistically improve the outcome of ICBs [13,14,15]. Similarly, pre-clinical data suggest that PTA enhances ICB outcomes by modifying the tumor microenvironment, promoting tumoral antigen presentation, and stimulating the innate immune system after inducing oncolysis [16,17,18,19]. In addition to direct effects on the targeted tumor, some studies have demonstrated the potential to induce an abscopal effect with broader systemic immunogenic responses in tumors located far from the targeted lesion [20,21]. While the literature on synergic abscopal effects remains scarce, the potential to safely combine targeted therapy with systemic immunotherapy bears potential that should be further explored.

The aim of our study was to report upon the safety and efficacy of combining ICBs and PTAs for patients with various metastatic neoplasms at a single tertiary cancer referral center. Understanding the safety profile of this combination treatment may validate broader clinical practice and expand future clinical trials. Efficacy outcomes of the combination therapy will be useful to standardize the application of local treatment in the context of ICB treatment.

## 2. Material and Methods

### 2.1. Study Design and Population

Institutional review board (Scientific Commission on Therapeutic Trials, Gustave Roussy) approval (no.: 2023-197) and patient informed consent were obtained for this retrospective, single-center cohort study. As we are a large tertiary cancer center, ICBs have been used in our institution since 2010. All patients ≥18 years of age who underwent concomitant PTA and ICB treatment from 2010 to 2021 were eligible for study inclusion. All medical files were sorted using a specific data search engine and warehouse developed for our electronic medical records system (Dr. Warehouse^®^). The search strategy was as follows: [Cryoablation OR Cryotherapy OR Radiofrequency OR micro-wave OR thermo-ablation OR electroporation] AND [immunotherapy OR pembrolizumab OR nivolumab OR ipilimumab OR atezolizumab OR durvalumab]. After this preliminary search, each medical file was reviewed to assess the chronicity of treatments. Inclusion criteria of the final cohort were established as ICB administration no more than 90 days prior to PTA (as exposure for most immunotherapy agents seems to still be present at 3 months) or 30 days after PTA (as most ablation-induced phenomena decrease drastically after this period).

### 2.2. Data Collection

Patients’ characteristics and their demographic and morphometric parameters were recorded, including age, sex, height, weight, and global health score (ECOG Performance Status) before PTA.

Oncological parameters were also collected, including the cancer type (primary tumor histology on biopsy samples), metastatic status, type of ICB used, time between PTA and ICB use, number of previous systemic treatment lines, cumulative time of ICB treatment before PTA, and any combination with chemo- or other locoregional targeted therapies such as radiotherapy or embolization.

PTA procedure parameters were recorded, including the ablation modality (cryoablation, radiofrequency ablation, or microwave ablation) and the PTA completeness (margins) based upon imaging at the end of the procedure. The lesion number, size, and location were also collected. The indication for PTA was allocated to 1 of the 3 following groups: oligo-persistence (objective response or disease control for all tumors but a few of them), oligo-progression (initially controlled metastatic tumor with recurrence or new progressive lesions limited to a few sites of disease), palliation of symptomatic lesions, or prevention of skeletal-related event (SRE). 

Lastly, the biological parameters including neutrophil-to-lymphocyte ratio (NLR) and lactate dehydrogenase (LDH) rate were collected before and within 30 days after PTA procedures to assess for general tumor and immune response.

### 2.3. Outcomes

The primary outcome was to assess the feasibility and safety of a combined treatment of PTA and ICBs. To that end, the technical success rate was defined as the percentage of treatment performed and completed as planned. Cancellation or abridgment of treatments was reviewed. Concerning safety, all procedural complications within 90 days following the PTA were collected. The incidence of irAEs within 1 year following PTA procedures was also assessed. Complications and/or adverse events were graded according to CTCAE v.5.0. Complications of grade 1 or 2 were considered minor complications, while grade 3 or greater were considered major complications.

Finally, and as a secondary outcome, the synergy of combination treatments was assessed by collection of OS, PFS, and _L_PFS and compared between subgroups using univariate and multivariate analysis. The intent was the evaluation of the synergistic effect within this combination cohort, particularly between different tumor types, sizes, and indications. No non-combination arm was used as a comparison.

### 2.4. Statistical Analysis

Continuous variables were expressed as median and interquartile range (IQR) or mean and standard deviation (SD), as appropriate. Categorical variables were expressed as frequencies and percentages (%). Survival data were estimated and graphically represented according to the Kaplan–Meier method and compared with the log-rank test. Cox’s proportional model was used to determine the hazard ratio regarding survival parameters between all subgroups in uni- and multivariate analysis. Missing values for LDHpre and LDHpost were considered as “missing at random” (MAR). Given their small number (about 20%), multiple imputation were performed for missing values using the R library «*MICE*». Two-sided *p* values ≤ 0.05 were considered statistically significant. All statistical analyses were performed using R-Studio version 4.1.1 (R Studio Inc., Boston, MA, USA).

## 3. Results

### 3.1. Study Population

In the *GR* medical database, we identified 320 patient files, of which 78 patients (41 males and 37 females) aged between 18 and 88 (median 61 [IQR 54–67]) met the inclusion criteria. The decision for PTA treatment was made in a multidisciplinary setting for all patients based upon tumor progression despite ongoing ICB treatment.

### 3.2. Patient, Tumor, and Treatment Characteristics

**Figure 1:**
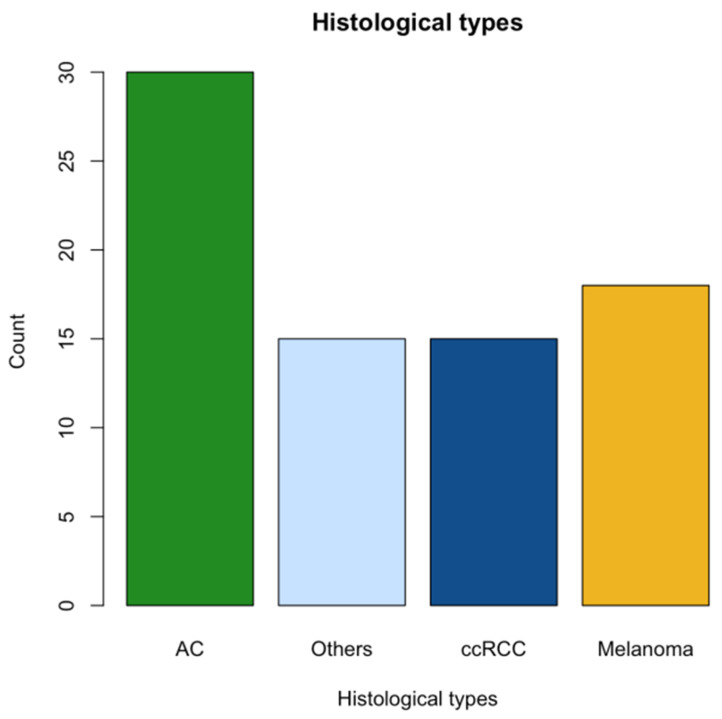
Bar chart representation of primary tumors’ histological distribution. *Adenocarcinoma (AC) was the most frequent histology (38%), followed by melanoma (23%) and cell carcinoma (ccRCC, 19%)*.

All patients had metastatic disease at the time of the PTA procedure. Most patients had a good global health status (ECOG PS < 2 (80%)). Furthermore, most patients (84%) had received no or a single previous line of systemic treatment before the ICB used at the time of PTA treatment. Table 1 summarizes most characteristics. The primary tumor location for most patients was lung (*n* = 23), skin (*n* = 20), or kidney (*n* = 17). The remaining 18 patients had primary tumors that included colorectal adenocarcinoma, hepatocellular carcinoma, and head and neck squamous cell carcinoma. The tumor histological subtypes are summarized in Figure 1.

The median time interval between PTA and a prior ICB injection was 14.5 days [IQR 6.25–23] and from the first ICB injection was 6 moths [IQR 2–11]. Similarly, the median time between PTA and the subsequent ICB injection was 15 days [IQR 10–21]. The majority of patients (67 patients) were only treated with immunotherapy including an anti-PD1 monoclonal antibody: pembrolizumab alone (*n* = 35/78, 45%), nivolumab alone (*n* = 24/78, 31%), or a combination of nivolumab and ipilimumab (*n* = 8/78, 10%). Only a few patients received ipilimumab alone (*n* = three patients) or anti-PDL1 antibodies such as atezolizumab or durvalumab (*n* = eight patients). A minority of patients underwent simultaneous treatment with two systemic therapies with different mechanisms: ICB and chemotherapy in eight patients (10%), and ICB and a targeted systemic therapy in seven patients (9%).

The indication for PTA was oligo-persistent disease in 23 patients (29%), oligo-progression in 11 patients (14%), palliation of symptomatic lesions, or prevention of SRE for the remaining 44 patients (56%).

CA and RFA were applied in 61% and 31% of cases, respectively, with microwave ablation (MA) applied in the remaining eight cases. Technical success was achieved in all cases (100%). The locations of metastasis predominantly comprised bone (43), lung (13), and liver lesions (7). Less frequent locations for ablations included the kidney, breast soft tissues, and musculature or retro peritoneum. In the vast majority of PTA procedures, a single lesion was targeted (81%) and, in the remainder, between 2 and 4 lesions were simultaneously ablated in a single session. The median lesion size ablated was 2.5 cm [IQR 2–3.75], with 35% less than 2 cm, 40% between 2 and 3 cm, and 26% greater than 3 cm. On immediate post-procedural imaging, PTA was considered complete in 62% of cases, with an ablation zone fully covering the lesion. Finally, 19 ablated lesions underwent an additional local treatment with either radiation therapy or percutaneous arterial embolization. In 17 patients, the lesion was also treated with radiation therapy (10 before PTA and 7 afterwards). In two patients, the lesion was treated with arterial embolization immediately before the PTA to improve the procedural bleeding risk. The lesion characteristics, PTA technique, associated locoregional treatments, and biological data are listed in Table 2.

### 3.3. Feasibility and Safety Analysis

All patients (100%) underwent treatment as planned, either ICB administration or PTA interventions. No cancellation or abridgement was recorded due to the combination of both therapeutics. 

Complications associated with PTA were recorded in 10 patients (13%). Nine of these procedure-related AEs were minor with CTCAE equal to or less than 2. In the one major complication (grade 3), intraprocedural hemoptysis during the lung ablation necessitated recovery and monitoring in intensive care for 3 days. The patient recovered without chronic sequelae and no additional intervention. 

**Figure 2:**
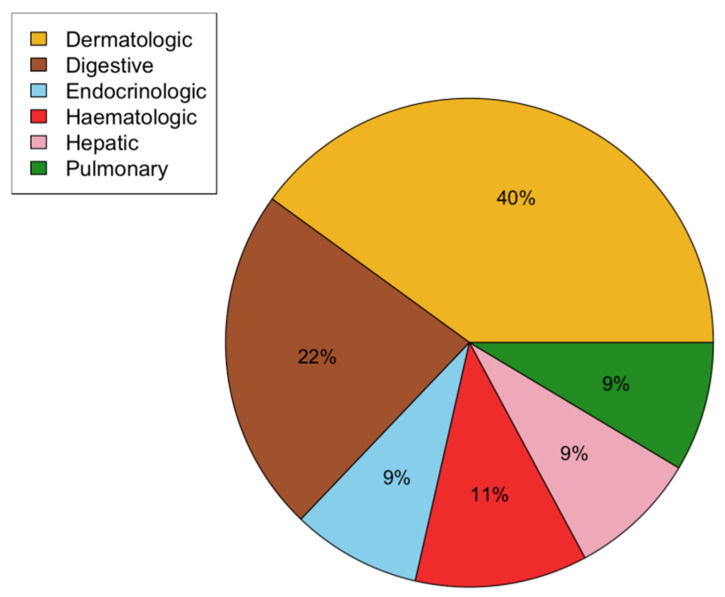
Graphical representation of irAE types sorted by organs affected. Types and frequencies of irAEs were consistent with known adverse events in the literature, with dermatological, digestive, and hematological side effects in most cases.

During the year following PTA, irAEs were observed in 44% of cases, with minor complications (CTCAE ≤ 2) reporting in most cases (67% of all irAEs) and major complications recorded in 11 cases (33% of all irAEs). The type of irAE listed by organ affected is displayed in Figure 2.

### 3.4. Outcome Analysis

**Figure 3:**
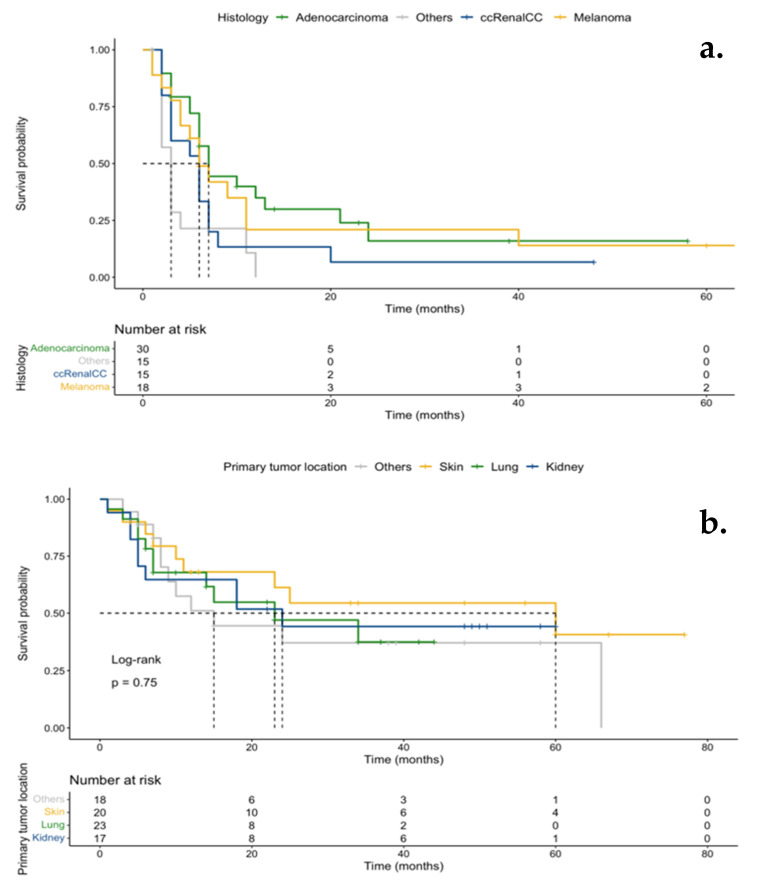
Kaplan–Meier OS according to primary tumor histology (**a**) and primary tumor location (**b**). No OS difference was observed according to tumor histology or primary tumor location. A trend for better survival was seen in patients treated for melanomas.

No difference in overall survival (OS) was demonstrated between PTA modality (*p* = 0.9), nor for the location or histologic type of the primary tumor (*p* = 0.75) (Figure 3a,b). A trend for better survival was noted among patients with melanomas. Lastly, no statistically significant difference in OS was seen between subgroups sorted by location of the ablated lesion (*p* = 0.88), although a slight increase was noted in the liver ablation subgroup (Supplemental Material Figure S1).

**Figure 4:**
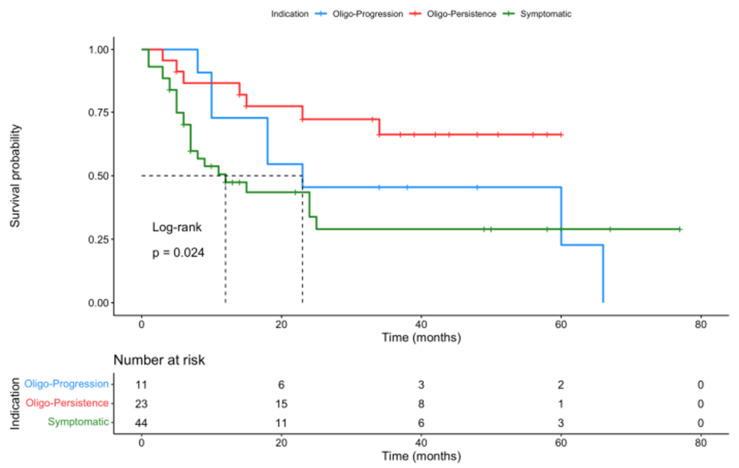
Kaplan–Meier OS graphical representation according to PTA indications. OS was significantly better in the oligo-persistence group compared to both other indications.

OS was significantly better in the oligo-persistence group (median survival not reached at time of analysis, [95%CI: 34 months—NR]) when compared to the oligo-progression subgroup (median survival time of 23 months, [95%CI: 18 months—NR]) and the palliation of symptomatic or prevention of SRE subgroup (median survival time of 12 months, [95%CI: 12 months—NR], *p* = 0.024) (Figure 4). In our multivariate analysis, the OS difference according to PTA indication was barely significant (*p* = 0.058).

PTA treatments for lesions located outside of the bone, liver, and lung were independently associated with a worse OS (*p* = 0.014), an impaired health condition (ECOG 2–3, *p* = 0.017), and treatment with ipilimumab alone (*p* = 0.004). This subcohort of 15 patients (19%) had PTA treatment in the kidney, breast, muscular, or retroperitoneal soft tissues (Supplemental Material Figure S2).

**Figure 5:**
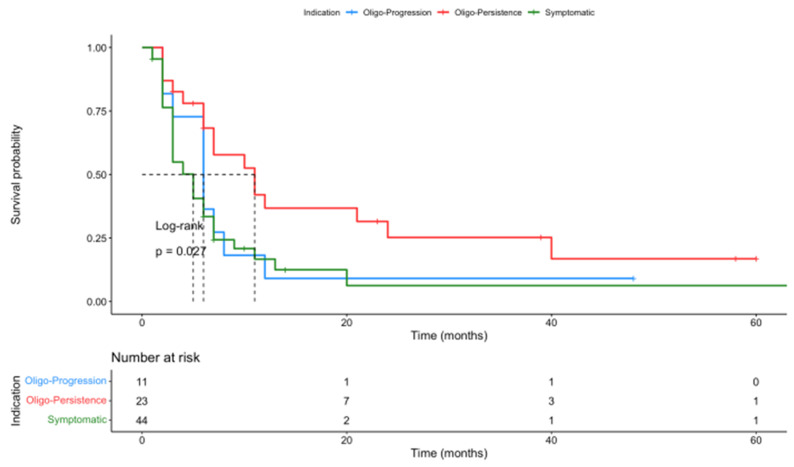
Kaplan–Meier PFS graphical representation according to PTA indications. PFS was significantly better in the oligo-persistence group compared to both other indications.

Progression-free survival (PFS) did not differ significantly between subgroups sorted by PTA technique (*p* = 0.82) or PTA location (*p* = 0.26) (Supplemental Material Figure S3). PFS was improved by primary histology, with statistical significance in favor of NSCLC and melanoma compared to RCC or other tumor types (sarcomas, colorectal cancer, hepatocellular carcinoma) (*p* = 0.019). A significant difference was also identified according to PTA indication, with better PFS in the oligo-persistence group (*p* = 0.027) (Figure 5).

In the multivariate analysis, the subgroups treated with ipilimumab or nivolumab + ipilimumab demonstrated worse PFS than patients treated with pembrolizumab. A greater number of ablated lesions was also significantly associated with a worse PFS (Supplemental Material Figure S4).

No significant difference in local PRS (_L_PFS) was present based upon PTA indication (*p* = 0.31) or PTA location (*p* = 0.38). Ablations of lung and liver lesions were noted to have slightly fewer local recurrences than bone ablations. After multivariate analysis, greater _L_PFS was also noted for lesions previously treated with other locoregional therapies (radiation therapy, surgery) and those treated with ipilimumab or nivolumab + ipilimumab (Supplemental Material Figure S5).

Lastly, there was no statistical significance associated with the biological parameters neutrophil-to-lymphocyte ratio (NLR) or lactate dehydrogenase (LDH) rate in our study.

### 3.5. Abscopal Effect Assessment

In four cases, a partial regression was observed for a lesion distant from the one treated with PTA within the next 6 months. In all of these cases, the patient had an overall stable tumor burden prior to PTA and there was no change in the ICB therapy before or after the PTA. For this reason, the response in distant lesions was attributed to systemic immunogenic stimulation or an “abscopal effect” related to PTA (Supplemental Data Figure S5).

## 4. Discussion

This retrospective study provides a real-world perspective on the methods and outcomes of a combined treatment of PTA and ICBs between 2010 and 2021. We aimed primarily to assess the safety of this combined therapeutic approach. The feasibility and safety profile were found to be excellent, with complications equivalent to those reported when each treatment was performed separately. Tumor progression and survival outcomes provide the practical conclusion that while no clear synergy was found for the combined approach, neither was there any demonstrable antagonistic effect. Hence, the combination of PTA and ICB treatments can be safely pursued for patients on ICB therapy with progressive disease.

Patient selection and termination of the indication for PTA treatment followed standard practices within our center. Indications for PTA included both curative and palliative intent. The three primary tumor types in our cohort (NSCLC, melanoma, and RCC) are typically treated with immunotherapy [1,2]. Pembrolizumab was the most commonly administered ICB. This is in keeping with current standards of practice, as pembrolizumab is an approved treatment for all three of these tumor types. In terms of the clinical presentation for PTA, patients had few comorbidities, few previous lines of systemic treatment, and a preserved performance status (ECOG < 2). Moreover, most lesions were solitary and small in size, both factors that have independently proven to provide a low risk of local recurrence after PTA. 

The technical approach to PTA in these cases was consistent with our normal clinical practice. While the success rate of PTA procedures was 100%, the completeness of ablation (i.e., including the entire tumor and with margins of at least 5 mm) was not consistent. This variability in the technical goal was in line with our regular clinical practice. For a PTA indication of local tumor control, technical methods are pursued to obtain complete margins. On the contrary, ablation without complete margins might be acceptable for PTA indications of pain palliation or prevention of skeletal-related events. The 5 mm cut-off is now well-accepted in the literature as the minimum margin to avoid local tumor recurrence. Increasingly powerful tools are now available, including algorithms for calculating and predicting these ablation margins, to improve the reproducibility and reliability of PTA [22].

The safety profile of combination PTA and ICBs was found to be excellent. Adverse events were found in comparable frequency and severity to those reported in the literature for each treatment separately [12,23,24]. The postulation that PTA might adversely boost the systemic immune-modulated side effects of ICBs was not observed. There was no cancellation or postponement of ICBs after PTA, and we found no indication that the combination therapy predisposed the patient to more frequent or severe adverse events. That suggests that no delay of immunotherapy treatment by more than 15 days is required in combination with PTA.

In both single variable and multivariate analysis, the indication for PTA treatment proved to be the parameter significantly associated with increased OS and PFS, and we found that the oligo-persistent patient subgroup received a tangible benefit. This finding is in keeping with the situation when PTA is pursued alone, without the use of ICB therapies, and may be related to patient selection for less aggressive tumors. This difference in survival can potentially be explained by the mechanisms of resistance to immunotherapy that differ between oligo-persistent and oligo-progressive patients. We assume that oligo-persistent disease originates from the primary resistance of a few metastases, mainly due to their locations, the so-called “immune sanctuaries”, whereas oligo progressive disease corresponds to locally acquired resistance either by clonal selection or by local modulation of immunity, and, therefore, has a poorer prognosis. This observation does not provide definitive evidence that the combination of PTA with ICBs portends a better or worse prognosis. Rather, this observation confirms that the selection of patients who have less aggressive tumors with solitary nodules that are completely ablated can convey an outcome advantage, regardless of whether the patient is under immunotherapy or not. 

Other factors that were analyzed for outcome effect included primary tumor type, metastatic lesion location, and ablation modality. Neither the primary tumor type nor the location of metastasis significantly affected OS or PFS. Again, this seems in keeping with known clinical practice for PTA alone, which suggests that complete ablation with sufficient margins is more critical to outcomes than tumor type or location. For melanoma and liver metastases, the subtle trend of improved outcomes may be explained by patient selection bias as these patients often fell into the oligo-persistence subgroup. Alternatively, we cannot discount that the subtle trend may indicate an increased susceptibility of melanoma and liver metastases to either PTA alone, ICB alone, or the combination. Some studies have noted that liver lesions are less responsive to ICB treatment and postulated that the liver may represent an immune sanctuary [25,26]. Another point to consider is the heterogeneous perfusion gradient present within tumors, especially when they are large with necrotic and other solid parts. This variability influences the size and shape of ablation zones, as well as the local bioavailability of the checkpoint blockers used [27]. Because of the heterogeneity of tumor histologies in our cohort, it is difficult to draw conclusions about which tumors are most favorable to combined treatment with PTA and ICI; instead, future studies on more homogeneous or comparative cohorts are needed to answer this question.

Regarding the treatment of bone metastases, a separate discussion is warranted to consider the factors that might explain the subtle, non-statistical trend of decreased PFS and _L_PFS. Similar to our explanation for the improved trend for melanoma and liver metastases, the converse can reasonably be applied to explain the decreased trend for bone metastases. In the approach to bone lesions, the primary clinical indication was often pain. Osseous metastases are frequently located in the vertebral column or near major pelvic joints. From a technical perspective, ablation with complete margins is often not pursued in these locations due to a high risk of permanent and debilitating damage to nearby nervous-critical structures. Given the ablation precaution in these locations, a common practice in our institution is to coordinate a combined locoregional treatment of PTA with radiation therapy to maximize the combined ablative response while decreasing procedural risk to these critical structures [28]. This practice explains the observation that the majority of PTAs for bone metastases were considered technically incomplete (37%), and it also explains the frequency of a combined radiation therapy + PTA treatment in this subset of patients (22%). Hence, the overall decrease in complete tumor ablation for bone metastases may be directly related to the non-statistically significant trend for a decreased in OS and PFS.

A synergistic effect of PTA and ICB therapy was considered, but without us finding a clear benefit or a definitive negative association. We reviewed the outcomes for PTA of multiple lesions, large lesions, and incomplete ablations to consider whether systemic administration of ICBs may have augmented the outcomes [11,29]. In the case of incomplete PTA, our results did not confirm any clinical suggestion that a combination of PTA and ICB treatment provided a significant benefit. Indeed, in keeping with PTA treatment alone, a technically complete PTA was associated in multivariate analysis with a better _L_PFS. Furthermore, the combination of PTA with an ICBs did not appear to significantly influence OS or PFS, either in a positive or negative manner. The lack of a positive association suggests that the use of an ICB is not sufficient to compensate for the higher likelihood of progression in patients with multifocal metastatic disease, nor for large or incompletely treated lesions. While this might initially be discouraging, the absence of a negative association addresses the concern that ICB treatment becomes less effective after incomplete PTA due to an acquired immune resistance [30]. This outcome was not found in our cohort and supports the use of PTA in the clinical setting of pain palliation and prevention of skeletal-related events, namely indications in which the entire tumor might not be safely ablated in its entirety. 

The prospect of an abscopal effect with the application of combination therapies is often optimistically raised [31], similarly to the combination of radiotherapy and immunotherapy [32]. In our study, four patients did demonstrate a partial regression of a lesion distant from the lesion treated with PTA. This occurred within 6 months of the PTA and could not be attributed to any changes in ICB therapy or other locoregional treatments. The physiopahtology underlying this abscopal effect following thermoablation can be explained by exposure of the immune system, and, in particular, dendritic cells, to intratumoral antigens after tumor cells have been destroyed by ablation. While this is a heartening observation, no definitive evidence exists to confirm the exact reason for this response. In a literature review, some pre-clinical data suggested that cryoablation may have a better immunogenic potential than radiofrequency ablation or microwave ablation, as there is less deterioration of intracellular antigens and organelles after cryoablation [17,18,19]. In our study, there was no significant difference observed in OS or PFS according to PTA modality, which may be due to lack of power of our study or to a real absence of correlation. 

The limitations of our retrospective study include a relatively small cohort, with only 78 patients treated over the course of 11 years. Furthermore, there was no comparison arm with PTA alone or ICB therapy alone. In addition, there was high variability among the tumor characteristics and treatments. Neither the metastatic location, lesion size, ablation modality, nor the frequency, dose, or specific ICB medication was standardized. Nonetheless, if we consider, for example, that treatment with anti-CTLA4 (ipilimumab) either alone or combined with nivolumab is now associated with a worse OS, PFS, and _L_PFS [33], then while the number of patients treated with ipilimumab in our cohort was small (*n* = 3.4%), they still provide an example that not all ICB therapies are equal. Lastly, several of our patients also had concomitant treatment with other locoregional treatments, including radiation therapy or arterial embolization. This will have induced additional selection bias, as these tumors may possess an acquired resistance or a more aggressive phenotype [34]. All of these limitations are acknowledged and yet we wish to highlight that our study design was meant to report and evaluate the general safety profile of PTA and ICBs in the real-life pragmatic clinical approach of a tertiary cancer center.

## 5. Conclusions

In summary, our retrospective review suggests that PTA and ICBs can be safely combined according to the independent indications of each treatment. Although no definitive positive synergy was demonstrated in our cohort, no negative outcomes of the combination were observed either. Several pre-clinical studies suggest that such a synergy may exist due to the stimulation of an immune response after PTA in the setting of ICB treatment, and scientific rationales have been suggested [20,21,22,23]. Randomized clinical studies are ongoing to test the hypothesis that combined treatments translate into a tangible and reproducible benefit (NCTs: CRYOMUNE, NCT: 04339218; NIVOLEP, NCT: 036306; AB-LATE 02 NCT: NCT04727307, etc.) [35,36]. Again, as these studies are ongoing and the field of immunotherapy is rapidly evolving, the intent of our focused study was primarily to report the excellent safety profile of combination therapy with PTA and ICBs over our broad 11-year experience.

## Figures and Tables

**Table 1:** Patient characteristics and oncological history.

Patient Characteristics [*n* (%) or Median (IQR)]	N = 78
Demographic and morphometric data	
Age (years)	61 (54–67)
Sex	
Male	41 (53)
Female	37 (47)
Weight (kg)	70 (57–85)
Height (cm)	171 (165–178)
ECOG performance status	
0–1	52 (80)
2–3	16 (20)
Primary tumor location	
Lung (NSCLC)	23 (29)
Skin	20 (26)
Kidney	17 (22)
Other	18 (23)
Metastasis location	
Bone	50 (64)
Lymphadenopathy	40 (51)
Lung	35 (45)
Liver	19 (24)
ICB therapy	
Pembrolizumab	35 (45)
Nivolumab	24 (31)
Nivolumab + ipilimumab	8 (10)
Ipilimumab	3 (4)
Other	8 (10)
Other systemic treatment	
None	63 (81)
Chemotherapies	8 (10)
Targeted therapies (TKI, anti-EGFR)	7 (9)

**Table 2:** Percutaneous ablation treatment characteristics.

PTA Characteristics [*n* (%) or Median (IQR)]	N = 78
Indication	
Oligo-persistence	23 (29)
Oligo-progression	11 (14)
Symptomatic lesions or prevention of SRE	44 (56)
Ablation technique	
CA	51 (65)
RF	24 (31)
MWA	3 (4)
PTA location	
Bone	43 (55)
Lung	13 (17)
Liver	7 (9)
Others	15 (19)
Previous treatment of ablated lesion	
RT	6 (8)
Surgery	4 (5)
None	68 (87)
Number of ablated lesion(s) during PTA	
1	63 (81)
2–4	15 (19)
Lesion size (cm)	
0–2	27 (35)
2–3	31 (40)
≥3	20 (26)
PTA considered technically complete	
Yes	48 (62)
No	29 (37)
Associated locoregional treatment	
Radiation therapy	17 (22)
Embolization	5 (6)
None	56 (72)
Biological data	
NLR pre-ablation	2.9 (1.9–5.3)
NLR post-ablation	2.6 (1.7–4.2)
LDH pre-ablation (U/L)	200 (175–222)
LDH post-ablation (U/L)	224 (185–277)

## Data Availability

The data presented in this study are available on request from the corresponding author (accurately indicate status).

## Supplementary Materials

The following supporting information can be downloaded at: https://www.mdpi.com/article/10.3390/cancers16050855/s1, Figure S1: Kaplan Meier OS according to PTA location; Figure S2: Updated OS by prespecified and post hoc exploratory subgroups; Figure S3: Updated PFS by prespecified and post hoc exploratory subgroups; Figure S4: Updated _L_PFS by prespecified and post hoc exploratory subgroups; Figure S5: Abscopal effect after bone cryoablation in metastatic NSCLC.

## Abbreviations

CA	Cryoablation
CTCAE	Common Terminology Criteria for Adverse Events v5.0
ICB	Immune checkpoint blocker
IQR	Interquartile ratio
irAEs	Immune-related adverse events
LDH	Lactate dehydrogenase
NLR	Neutrophil-to-lymphocyte ratio
OS	Overall survival
PD1	Programmed cell death 1
PD(L)1	Programmed cell death ligand 1
_L_PFS	Local progression-free survival
PFS	Progression-free survival
PTA	Percutaneous thermal ablation
RFA	Radiofrequency ablation
SD	Standard deviation
SRE	Skeletal-related events
