# Supplementary material for: Thermal Ablation Combined with Immune Checkpoint Blockers: A 10-Year Monocentric Experience
Source: Cancers (Basel). 2024 Feb 21;16(5):855. doi: 10.3390/cancers16050855 (PMC10931410; doi:10.3390/cancers16050855)
Supplement: Supplementary file 1 [file cancers-16-00855-s001.zip › cancers-2832323-supplementary.pdf]

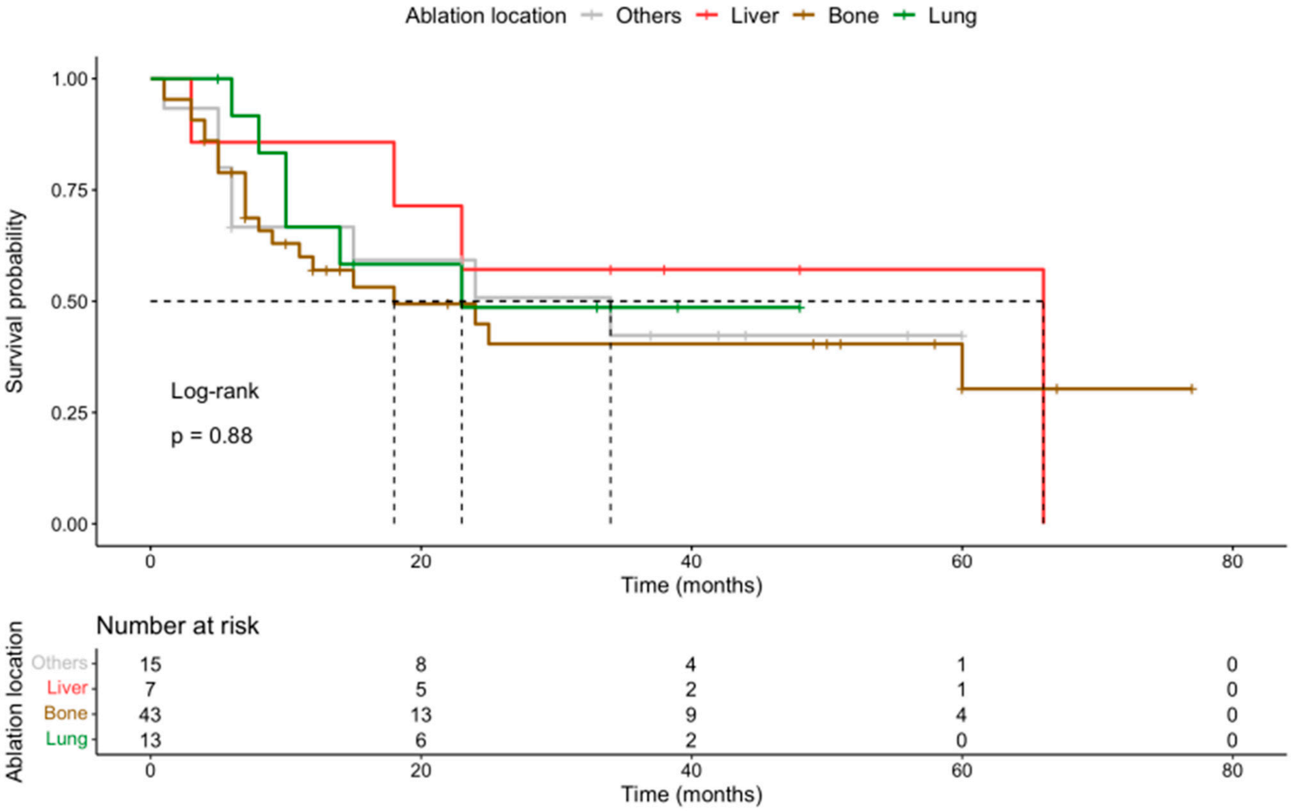

Figure S1. Kaplan Meier OS according to PTA location.

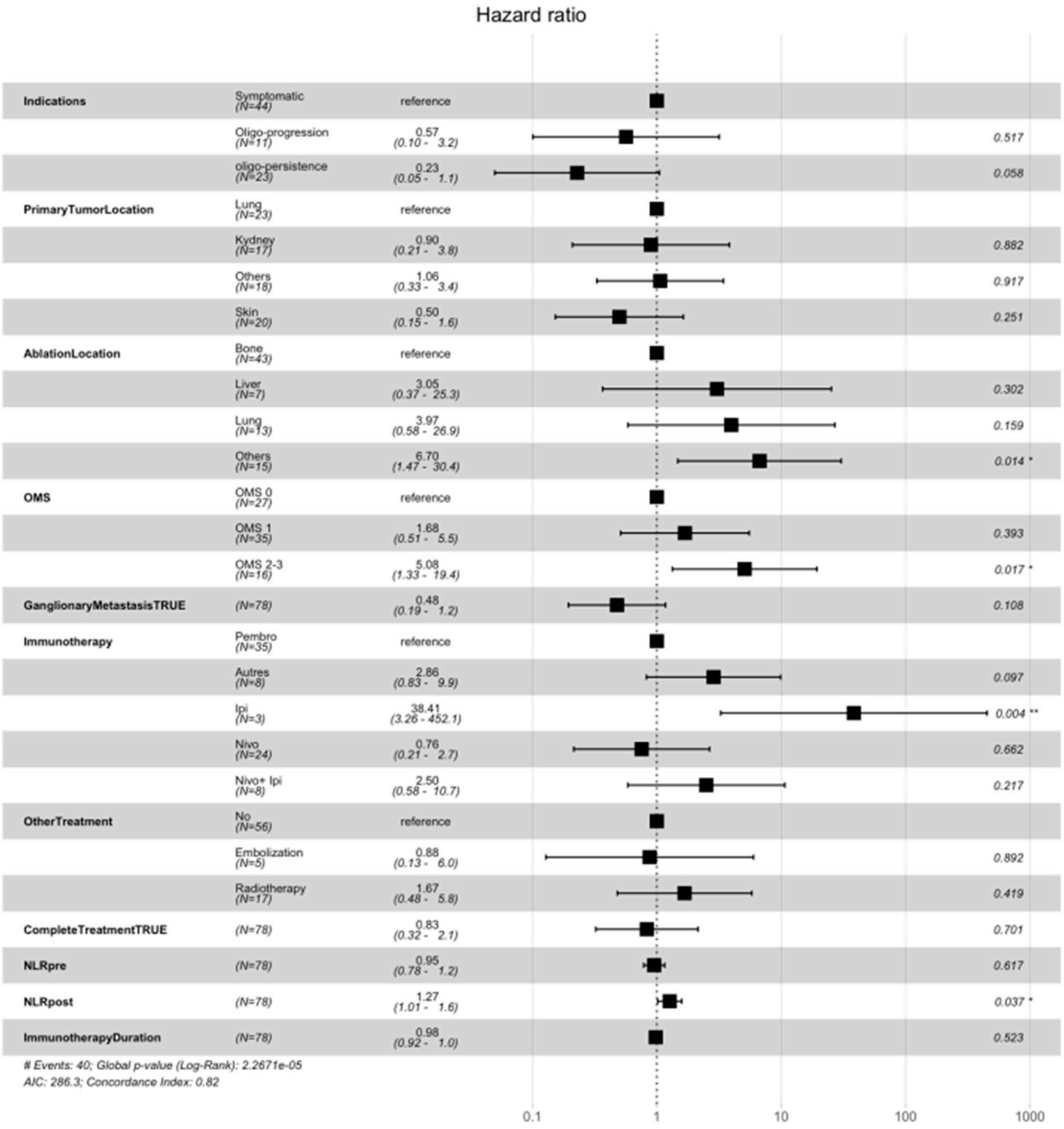

Figure S2. Updated OS by prespecified and post hoc exploratory subgroups.

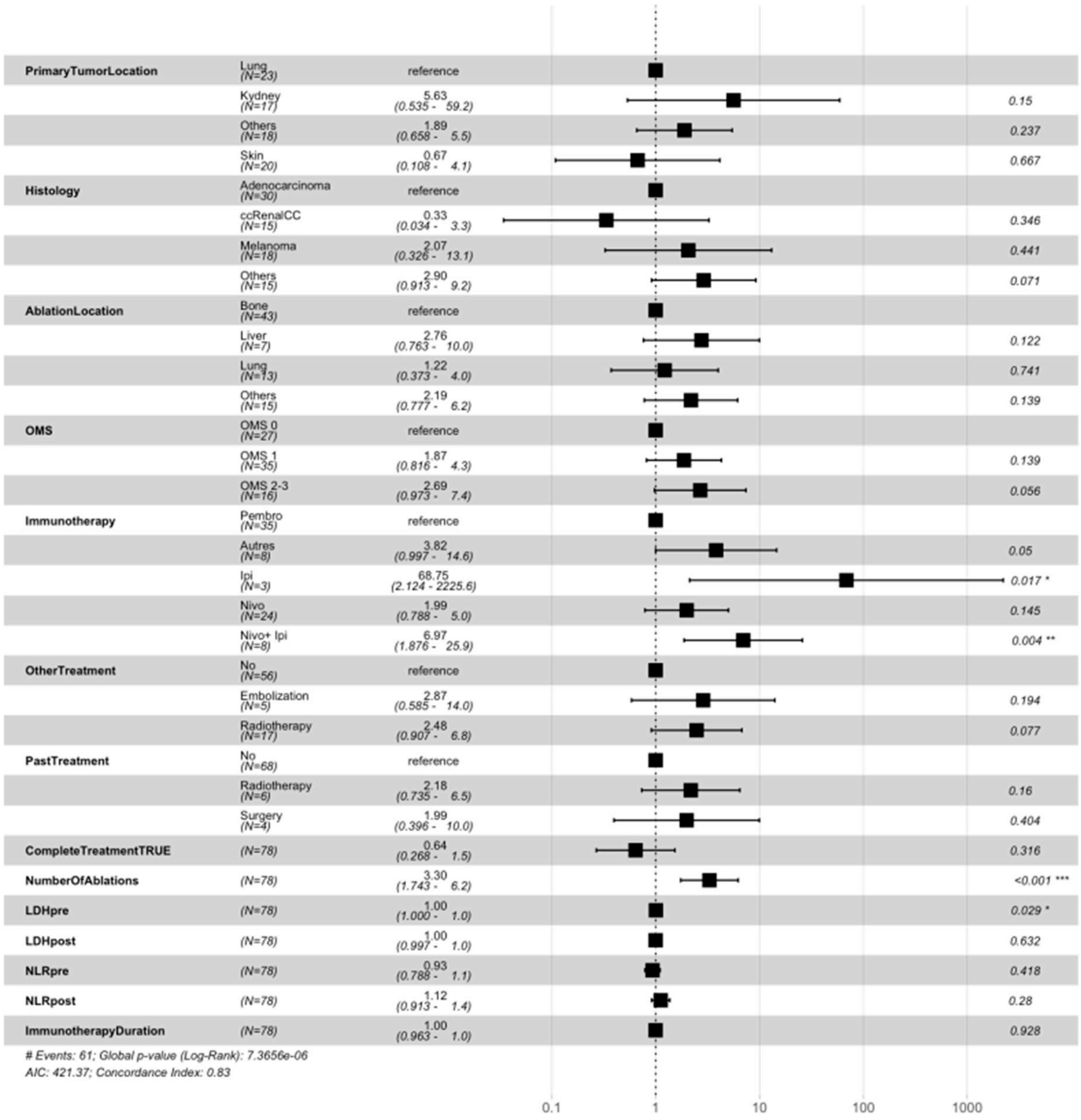

Figure S3. Updated PFS by prespecified and post hoc exploratory subgroups.

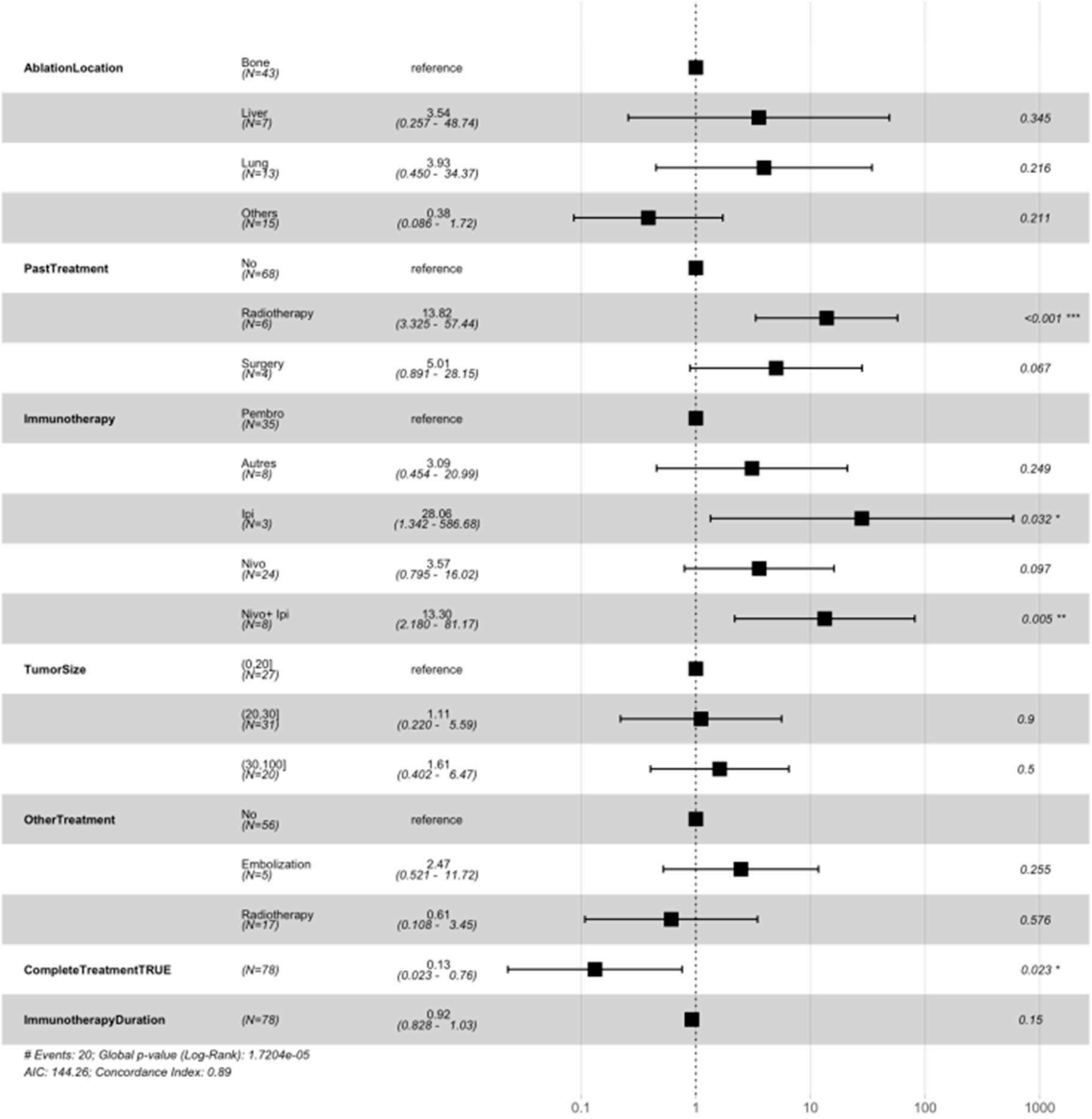

Figure S4. Updated lPFS by prespecified and post hoc exploratory subgroups.

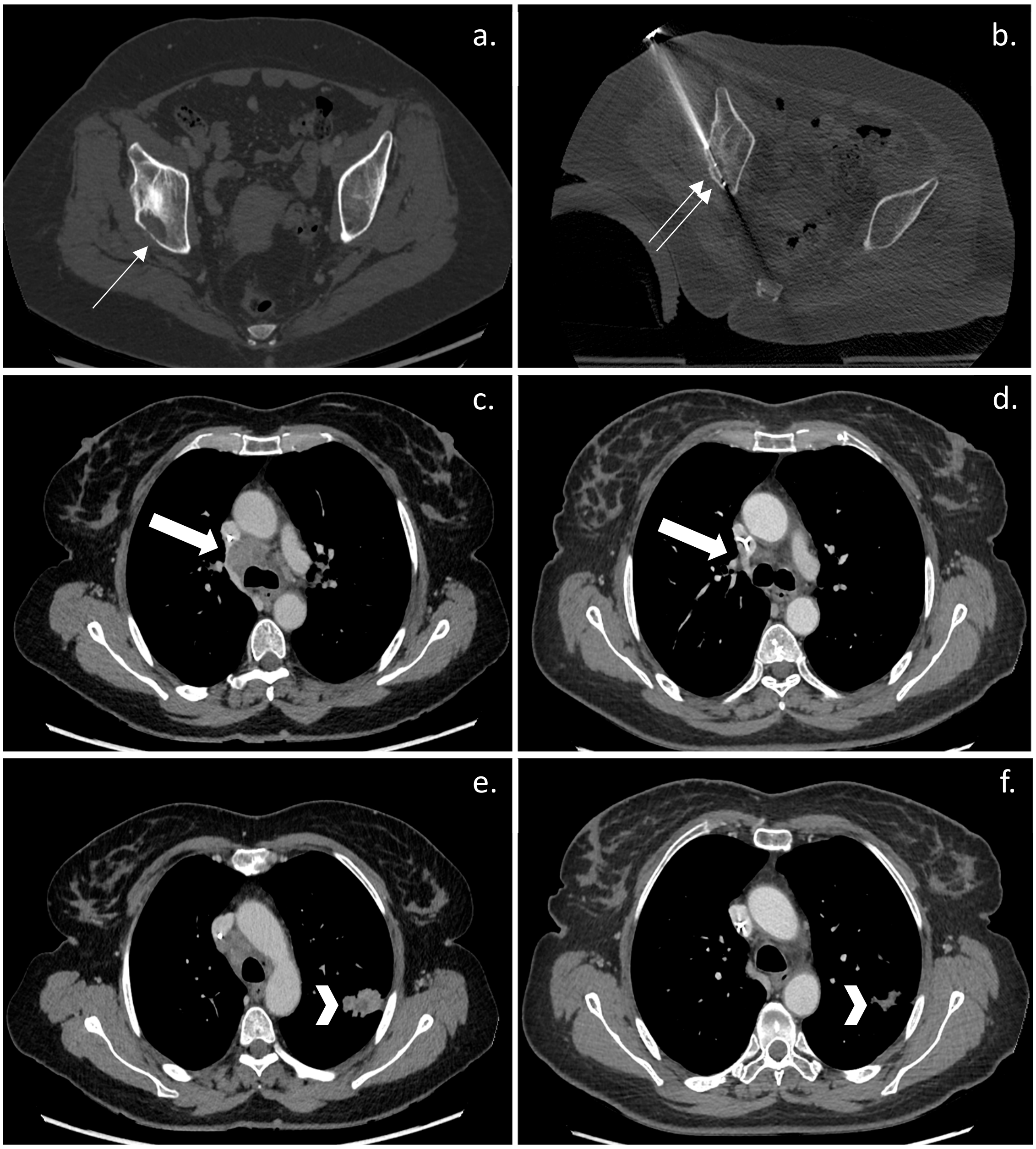

Figure S5.Abscopal effect after bone cryoablation in metastatic NSCLC. *Legend (Supp. Data. Fig. 5) –* 43 year-old patient with metastatic stage IV NSCLC treated with combine chemotherapy and immunotherapy (pembrolizumab), had stable disease for 4 months before ablation. Painful secondary lesion of the pelvic bone (*image a.* single thin white arrow) was referred for cryoablation and cementoplasty in a palliative approach to prevent SRE and treat the pain (*image b.* double thin white arrows). Systemic control by CT-scan two months after ablation showed shrinkage of secondary lesions distant from the ablation zone without changing systemic treatment, well seen on mediastinal lymph nodes.

KEY POINTS

(1) Question: What are the feasibility and safety of concomitant treatment with percutaneous thermal ablation (PTA) and immune checkpoint blockers (ICB) in general oncologicpopulation?

Findings: In this retrospective cohort study that included 78 patients, the feasibility was 100%, no treatment was contraindicated due to the combination. 13% experienced procedure-related complications (90% grade 1-2), 44% experienced an irAE (86% grade 1-2). No increase of complication was seen due to combination compared with each treatment alone.

Meaning: Concomitant treatment of PTA and ICB is feasible and safe without restriction in day to day practice

(2) Question: Which population benefit the most from comnination PTA and ICB therapy?

Findings: In this study, the only factor statistically associated with better OS and PFS was the ablation indication, favoring oligo-persistence ($P = 0.02$)

Meaning: Patients in oligo-persistent situation seem to be the best indication for combined PTA and ICB treatment.
